# Changes in stroke volume induced by passive leg raising in spontaneously breathing patients: comparison between echocardiography and Vigileo™/FloTrac™ device

**DOI:** 10.1186/cc8195

**Published:** 2009-12-07

**Authors:** Matthieu Biais, Lionel Vidil, Philippe Sarrabay, Vincent Cottenceau, Philippe Revel, François Sztark

**Affiliations:** 1 Service d'Anesthésie Réanimation 1, Hôpital Pellegrin, CHU Bordeaux, Place Amélie Raba-Léon, 33076 Bordeaux Cedex, France

## Abstract

**Introduction:**

Passive leg raising (PLR) is a simple reversible maneuver that mimics rapid fluid loading and increases cardiac preload. The effects of this endogenous volume expansion on stroke volume enable the testing of fluid responsiveness with accuracy in spontaneously breathing patients. However, this maneuver requires the determination of stroke volume with a fast-response device, because the hemodynamic changes may be transient. The Vigileo™ monitor (Vigileo™; Flotrac™; Edwards Lifesciences, Irvine, CA, USA) analyzes systemic arterial pressure wave and allows continuous stroke volume monitoring. The aims of this study were (i) to compare changes in stroke volume induced by passive leg raising measured with the Vigileo™ device and with transthoracic echocardiography and (ii) to compare their ability to predict fluid responsiveness.

**Methods:**

Thirty-four patients with spontaneous breathing activity and considered for volume expansion were included. Measurements of stroke volume were obtained with transthoracic echocardiography (SV-TTE) and with the Vigileo™ (SV-Flotrac) in a semi-recumbent position, during PLR and after volume expansion (500 ml saline). Patients were responders to volume expansion if SV-TTE increased ≥ 15%.

**Results:**

Four patients were excluded. No patients received vasoactive drugs. Seven patients presented septic hypovolemia. PLR-induced changes in SV-TTE and in SV-Flotrac were correlated (r^2 ^= 0.56, *P *< 0.0001). An increase in SV-TTE ≥ 13% during PLR was predictive of response to volume expansion with a sensitivity of 100% and a specificity of 80%. An increase in SV-Flotrac ≥16% during PLR was predictive of response to volume expansion with a sensitivity of 85% and a specificity of 90%. There was no difference between the area under the ROC curve for PLR-induced changes in SV-TTE (AUC = 0.96 ± 0.03) or SV-Flotrac (AUC = 0.92 ± 0.05). Volume expansion-induced changes in SV-TTE correlated with volume expansion-induced changes in SV-Flotrac (r^2 ^= 0.77, *P *< 0.0001). In all patients, the highest plateau value of SV-TTE recorded during PLR was obtained within the first 90 s following leg elevation, whereas it was 120 s for SV-Flotrac.

**Conclusions:**

PLR-induced changes in SV-Flotrac are able to predict the response to volume expansion in spontaneously breathing patients without vasoactive support.

## Introduction

Fluid is administered to critically ill patients in order to increase cardiac preload and cardiac output (CO). Studies have shown that about 50% of critically ill patients do not exhibit the desired effect [1]. Static indices and routine clinical variables are known to be of little value in discriminating between patients who will and those who will not respond to volume expansion (VE) [2]. In contrast, dynamic indices based on cardiopulmonary interactions and variation in left ventricular stroke volume (SV) are able to predict adequately the individual response to fluid loading in mechanically ventilated patients [3-9]. However, these indices appear inaccurate in spontaneously breathing patients because they strongly depend on respiratory status, which is not controlled in this case.

Passive leg raising (PLR) is a simple reversible maneuver that mimics rapid fluid loading. It transiently and reversibly increases venous return by shifting venous blood from the legs and the splanchnic reservoir to the intrathoracic compartment [10-15]. PLR increases the right cardiac preload. If the right ventricle is preload-responsive, an increase in right CO and left ventricular filling is observed. As a result, PLR may finally induce an increase in SV, depending on the degree of left ventricular preload reserve. On the contrary, if the right and/or the left ventricle are not preload-responsive, no increase in left ventricular SV is expected. Thus, PLR has been proposed as a test to detect fluid responsiveness in critically ill patients [10,12,13].

PLR has been validated to predict fluid responsiveness, but it requires the determination of CO with a fast-response device, because the hemodynamic changes may be transient [13,16]. The techniques available at present are transthoracic echocardiography, esophageal Doppler, transpulmonary thermodilution (PiCCOplus^®^, Pulsion Medical Systems™, Munich, Germany) and transthoracic Doppler ultrasonography (USCOM^®^; Uscom Ltd., Sydney, Australia) [10,12,13,17].

The recently introduced Vigileo™ monitor, which allows continuous CO monitoring, is based on the analysis of the systemic arterial pressure wave and does not require pulmonary artery catheterization or calibration with another method [18]. The aims of the study were to compare changes in SV induced by PLR obtained with the Vigileo™ and transthoracic echocardiography and to compare their ability to predict fluid responsiveness in spontaneously breathing patients.

## Materials and methods

### Patients

After approval by the local ethics committee and obtaining written informed consent, we included 34 patients with spontaneous breathing activity, equipped with an arterial catheter and a central venous catheter, and for whom the decision to give fluid was taken by the physician. This decision was based on the presence of at least one clinical or biological sign of inadequate tissue perfusion defined as (i) systolic blood pressure below 90 mmHg (or a decrease >50 mmHg in previously hypertensive patients), (ii) oligoanuria (urine output <0.5 mL/kg/hr for >2 hours) or biological signs of acute renal failure, (iii) tachycardia (heart rate >100 beats/min), or (iv) presence of skin mottling.

Exclusion criteria were: unsatisfactory cardiac echogenicity, increase in intra-abdominal pressure suspected by clinical context and examination, patients younger than 18 years, body mass index greater than 40 kg/m^2 ^or less than 15 kg/m^2^, aortic valvulopathy, mitral insufficiency greater than grade 2, mitral stenosis, or intracardiac shunt.

### Hemodynamic monitoring

#### Vigileo™ monitor

A dedicated transducer (FloTrac™, Edwards Lifesciences, Irvine, CA, USA) was connected to the radial arterial line on one side and to the Vigileo™ System (Edwards Lifesciences, Irvine, CA, USA) on the other side. The system, which enables the continuous monitoring of arterial pressure, CO (cardiac output obtained with Vigileo device (CO-Flotrac))and SV (stroke volume obtained with Vigileo device (SV-Flotrac)), needs no external calibration and provides continuous CO measurements from the arterial pressure wave. The Vigileo™ (Software version 1.14) analyzes the pressure waveform 100 times per second over 20 seconds, capturing 2000 data points for analysis and performs its calculations on the most recent 20 seconds of data. The device calculates SV as k × pulsatility, where pulsatility is the standard deviation of arterial pressure over a 20-second interval, and k is a factor quantifying arterial compliance and vascular resistance. k is derived from a multivariate regression model including (i) Langewouter's aortic compliance [19], (ii) mean arterial pressure (MAP), (iii) variance, (iv) skewness and (v) kurtosis of the pressure curve. The rate of adjustment of k is one minute (Software 1.14).

#### Echocardiographic measurements

Doppler echocardiography was performed by the same operator (MB) using a standard transthoracic probe (P4-2, Siemens Medical System, Malvern, PA, USA) and a dedicated unit (Acuson CV-70, Siemens Medical System, Malvern, PA, USA). Stroke volume obtained with transthoracic echocardiography (SV-TTE) was calculated as the product of the aortic valve area by the velocity time integral of aortic blood flow (VTIAo). Using the parasternal long axis view, the diameter of the aortic cusp and the aortic valve area was calculated (π diameter^2^/4). As the diameter of the aortic orifice is assumed to remain constant in a given patient, the diameter was measured once at baseline. Using the apical five-chamber view, the VTIAo was computed from the area under the envelope of the pulsed-wave Doppler signal obtained at the level of the aortic annulus. The VTIAo value was averaged over five consecutive measurements. Cardiac output obtained with transthoracic echocardiography (CO-TTE) was calculated as the product of heart rate (HR) by SV-TTE. The operator was unaware of SV and CO-Flotrac values.

Left ventricular ejection fraction was measured using Simpson's biplane method from the apical two- and four-chamber views.

#### Central venous pressure measurements

Central venous pressure (CVP) was determined at end-expiration and was averaged from three consecutive respiratory cycles.

#### Systemic vascular resistance calculation

Systemic vascular resistance (SVR) were calculated using the equation: SVR = (MAP-CVP) × 80/CO-TTE.

### Respiratory parameters

All patients were breathing spontaneously.

### Study design

A first set of measurements (HR, MAP, CVP, SV-FloTrac, VTIAo, left ventricular ejection fraction and aortic valve area) was obtained in the semi-recumbent position (45°; designated 'baseline'). Then, the lower limbs were lifted while straight (45°) with the trunk lowered in the supine position. The second set of measurements of MAP, CVP, HR, VTIAo (designated 'during PLR') was obtained during leg elevation, at the moment when VTIAo plateaued at its highest value. The stroke volume obtained with Vigileo device (SV-Flotrac) was recorded at the moment when it plateaued at its highest value. The body posture was then returned to the baseline position and a third set of measurements (MAP, CVP, HR, VTIAo and SV-FloTrac) was recorded (designated 'before VE'). Finally, measurements were obtained after VE, which was performed for 15 minutes with 500 ml saline (designated 'after VE').

### Statistical analysis

Results were expressed as mean ± standard deviation (SD) if data were normally distributed or median [25-75% interquartile range] if not. Patients were separated into responders (Rs) and non-responders (NRs) by change in SV-TTE of 15% or more and less than 15%, respectively, following the volume challenge [5,6,13]. Changes in hemodynamic parameters induced by changes in loading conditions were assessed using a non-parametric Mann-Whitney U-test or Wilcoxon rank sum test when appropriate. The Spearman rank method was used to test linear correlations. Receiver operating characteristic (ROC) curves were generated for PLR-induced changes in SV-TTE and PLR-induced changes in SV-FloTrac varying the discriminating threshold of each parameters, and area under the ROC curves (95% confidence interval (CI)) were calculated and compared [20].

SV-TTE and SV-Flotrac were compared using the Bland and Altman method [21]. Bias (mean difference between SV-TTE and SV-Flotrac) represents the systematic error between both methods. Precision (SD of the bias) is representative of the random error or variability between the different techniques. The limits of agreement were calculated as bias ± two SD, and defined the range in which 95% of the differences between the methods were expected to lie. The percentage error was calculated as the ratio of two SD of the bias to mean CO and was considered clinically acceptable if it was below 30%, as proposed by Critchley and Critchley [22].

A *P *value of less than 0.05 was considered to be statistically significant. Statistical analysis was performed using Statview for Windows, version 5 (SAS Institute, Cary, NC, USA) and Medcalc (software 8.1.1.0; Mariakerke, Belgium).

## Results

### Patient characteristics

Thirty-four patients were initially included. Four patients were excluded from analysis because of difficulties in transthoracic echocardiographic image analysis. The characteristics of the 30 patients finally studied are reported in Table 1.

**Table 1:** Patient characteristics

Characteristics	
Age (years)	55 ± 17
Gender M/F	21/9
Weight, kg	77 ± 20
Body mass index (kg/m^2^)	26 ± 5
Body surface area (m^2^)	1.89 ± 0.24
Reasons for fluid administration	
Septic hypovolemia	7
Non septic hypovolemia	23
Reasons for ICU admission	
Vascular surgery	13 (SH = 3)
Digestive surgery	12 (SH = 4)
Kidney transplantation	3 (SH = 0)
Brain injury	2 (SH = 0)

n = 30. F = female; ICU = intensive care unit; M = male; SH = septic hypovolemia.

Patients were included 1.4 ± 1.3 days after admission to the intensive care unit. No patients received beta-blockers. Every patient was breathing spontaneously. Nineteen patients (65%) were intubated and ventilated with pressure support (inspiratory pressure = 11 ± 3 cmH_2_O, end-expiratory pressure = 3 ± 2 cmH_2_O, fraction of inspired oxygen = 33 ± 7%). Eleven patients were not intubated.

No patient received vasoactive drugs. The decision to give fluid was made for low urine output (n = 14), tachycardia (n = 7), biological signs of acute renal failure (n = 4), mottling (n = 3), and low systolic blood pressure (n = 2).

Twenty patients were Rs to VE and 10 were NRs. The effects of PLR and VE on hemodynamic variables in Rs and NRs are shown in Table 2.

**Table 2:** Hemodynamic variables in responders and non-responders at baseline, during passive leg raising, before volume expansion and after volume expansion

	Baseline	During PLR	*P1*	Before VE	After VE	*P2*
HR (beats/min)						
*Responders*	78 (66-114)	78 (65-112)	NS	78 (66-109)	77 (65-109)	NS
*Non-responders*	78 (69-90)	78 (70-88)	NS	81 (69-90)	81 (70-91)	NS
MAP (mmHg)						
*Responders*	83 (71-96)	85 (77-97)	NS	84 (69-95)	98 (77-102)	0.0005
*Non-responders*	79 (73-91)	79 (69-85)	NS	75 (70-88)	79 (73-85)	NS
SV-TTE (ml)						
*Responders*	72 (61-85)	88 (74-102)	<0.0001	70 (58-85)	91 (74-105)	<0.0001
*Non-responders*	79 (72-95)	84 (76-91)	NS	77 (72-89)	82 (78-90)	0.02
SV-FloTrac (ml)						
*Responders*	73 (58-86)	88 (76-103)	<0.0001	72 (61-83)	90 (78-106)	<0.0001
*Non-responders*	80 (65-88)	79 (68-97)	NS	80 (65-86)	85 (67-98)	0.005
VTIAo (cm)						
*Responders*	21 (19-27)	27 (24-32)	<0.0001	22 (19-26)	29 (23-32)	<0.0001
*Nonresponders*	25 (21-27)	26 (24-27)	NS	22 (21-27)	25 (22-27)	0.02
CO-TTE (l/min)						
*Responders*	5.9 (4.8-7.2)	7.1 (5.8-9.1)	<0.0001	5.8 (4.7-7.1)	7.2 (6.1-8.8)	<0.0001
*Non-responders*	6.2 (6.1-6.6)	6.3 (6.1-7.7)	NS	6.1 (5.5-6.7)	6.5 (6.0-7.1)	0.02
CO-FloTrac (l/min)						
*Responders*	5.7 (4.5-7.7)	6.7 (5.7-9.5)	<0.0001	5.6 (4.6-7.8)	7.2 (6.1-9.5)	<0.0001
*Non-responders*	6.1 (5.4-6.4)	6.3 (5.2-7.5)	NS	5.9 (5.4-6.6)	6.4 (5.9-7.6)	0.005
SVR (dyn/s/cm^-5^)						
*Responders*	968 (806-1187)	805 (721-1082)	0.0002	960 (820-1275)	826 (702-1085)	0.0006
*Non-responders*	865 (807-1026)	810 (737-938)	NS	816 (781-1089)	820 (711-997)	NS
CVP (mmHg)						
*Responders*	5 (5-5)	8 (7-9)	<0.0001	5 (5-5)	9 (8-10)	<0.0001
*Non-responders*	9 (6-10)	10 (7-11)	0.01	9 (6-10)	10 (7-11)	0.01

CO-FloTrac = cardiac output obtained with Vigileo™ device; CO-TTE = cardiac output obtained with transthoracic echocardiography; CVP = central venous pressure; HR = heart rate; MAP = mean arterial pressure; PLR = passive leg raising; SV-Flotrac = stroke volume obtained with Vigileo™ device; SVR = systemic vascular resistance; SV-TTE = stroke volume obtained with transthoracic echocardiography; VE = volume expansion; VTIAo = velocity-time integral of aortic blood flow. *P1 *= during PLR values *vs *baseline values, *P2 *= after VE values *vs *before VE values.

### Effects of PLR and VE on changes in SV-TTE

**Figure 1:**
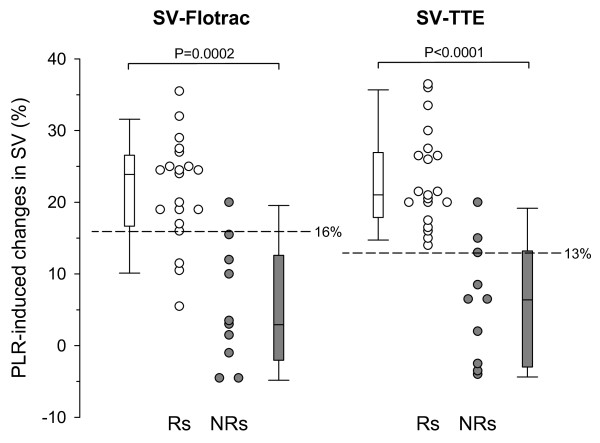
PLR-induced changes in SV-TTE and in SV-Flotrac in responders and non-responders. Box plots and individual values of passive leg raising (PLR)-induced changes in stroke volume measured with transthoracic echocardiography (SV-TTE) and with Vigileo™ (SV-Flotrac) in responders (Rs) and in non-responders (NRs).

In all patients, the effect of PLR on SV-TTE occurred in the first 90 seconds. Changes in SV-TTE induced by PLR were significantly greater in Rs than in NRs (*P *< 0.0001; Figure 1). In Rs, SV-TTE increased by 21 (18 to 27) % from baseline to PLR and by 28 (25 to 36) % from before VE to after VE. In NRs, SV-TTE increased by 6 (-3 to 13) % from baseline to during PLR and by 12 (1 to 14) % from before VE to after VE.

### Effects of PLR and VE on changes in SV-FloTrac

In all patients, the effect of PLR on SV-Flotrac occurred in the first 120 seconds. The changes in SV-Flotrac induced by PLR were significantly greater in Rs than in NRs (*P *= 0.0002). In Rs, SV-Flotrac increased by 24 (18 to 26) % from baseline to during PLR and by 25 (22 to 30) from before VE to after VE. In NRs, SV-Flotrac increased by 3 (-1 to 12) % from baseline to during PLR and by 7 (5 to 11) from before VE to after VE.

### Comparison between changes in SV-TTE and changes in SV-FloTrac

**Figure 2:**
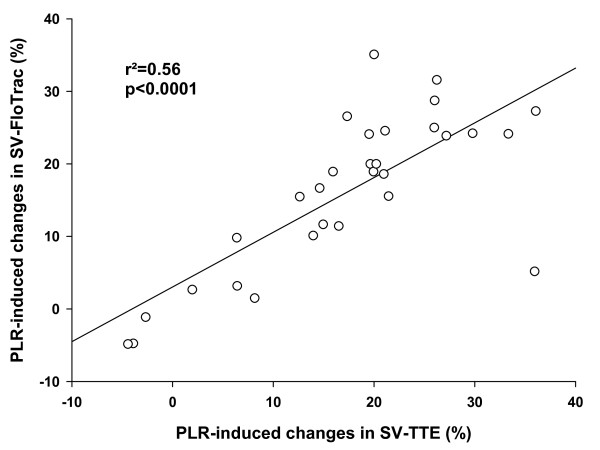
Relation between PLR-induced changes in SV-TTE and SV-Flotrac. Relation between passive leg raising (PLR)-induced changes in stroke volume measured with transthoracic echocardiography (SV-TTE) and with Vigileo™ (SV-FloTrac).

**Figure 3:**
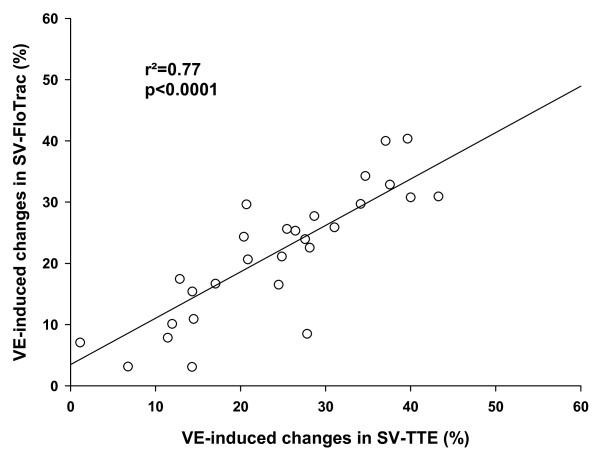
Relation between VE-induced changes in SV-TTE and SV-Flotrac. Relation between volume expansion (VE)-induced changes in stroke volume measured with transthoracic echocardiography (SV-TTE) and with Vigileo™ (SV-FloTrac).

The correlation between PLR-induced changes in SV-TTE and SV-Flotrac was r^2 ^= 0.56 (*P *< 0.0001) and the correlation between VE-induced changes in SV-TTE and SV-Flotrac was r^2 ^= 0.77 (*P *< 0.0001; Figures 2 and 3). After VE, the classification between Rs and NRs was similar using SV-TTE and SV-FloTrac in 29 patients (97%).

### Prediction of fluid responsiveness

#### PLR-induced changes in SV-TTE

An increase in SV-TTE induced by PLR of more than 13% predicted the response to VE (increase in SV-TTE ≥ 15% following VE) with a sensitivity of 100% (95% CI = 83 to 100) and a specificity of 80% (95% CI = 44 to 97). Two patients exhibited an increase in SV-TTE of more than 13% induced by PLR whereas they were NRs to VE.

#### PLR-induced changes in SV-FloTrac

An increase in SV-Flotrac induced by PLR of more than 16% predicted the response to VE (increase in SV-TTE = 15% following VE) with a sensitivity of 85% (95% CI = 62 to 97) and a specificity of 90% (95% CI = 56 to 98). Three patients did not exhibit an increase in SV-FloTrac of more than 16% during PLR whereas they were Rs to VE and in one patient, SV-FloTrac was more than 16% during PLR whereas he was NR to VE.

**Figure 4:**
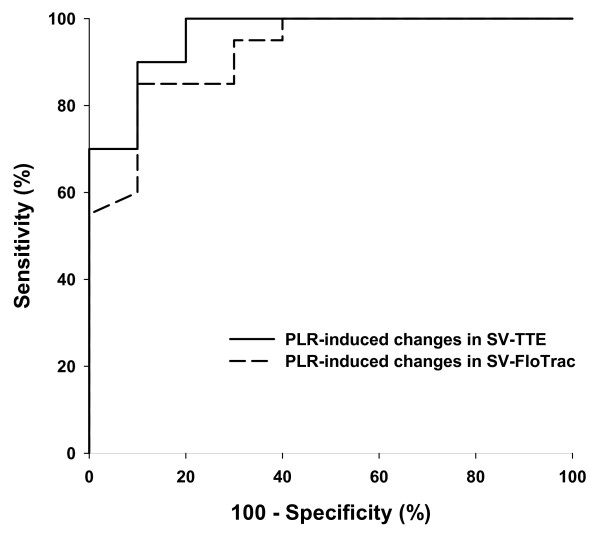
ROC curves for predicting response to volume expansion. Receiver operating characteristic (ROC) curves comparing the ability of passive leg raising (PLR)-induced changes in stroke volume measured with transthoracic echocardiography (SV-TTE) and with Vigileo™ (SV-Flotrac) to discriminate responders and non-responders following volume expansion.

There was no difference between the areas under the ROC curve for PLR-induced changes in SV-TTE (0.96 ± 0.03) or SV-Flotrac (0.92 ± 0.05; Figure 4)

### SV comparison

**Figure 5:**
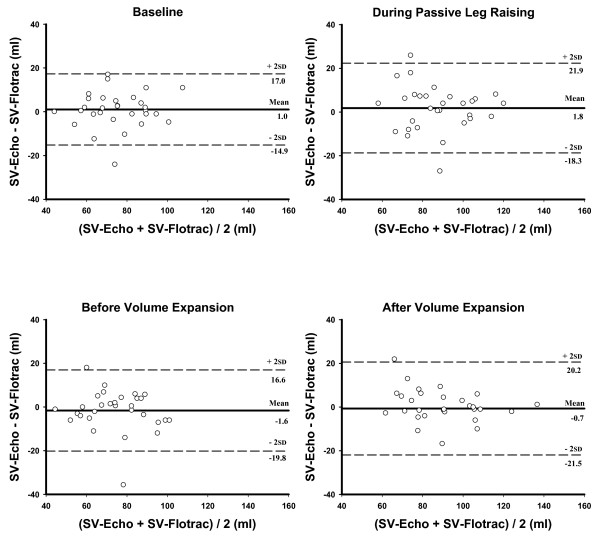
Comparison between SV-TTE and SV-Flotrac at baseline, during PLR, before VE and after VE. Bland-Altman plots between stroke volume measured by transthoracic echocardiography (SV-TTE) and by Vigileo™ (SV-Flotrac) at baseline, during passive leg raising, before and after volume expansion. The continuous lines show the mean difference (bias) and the dotted lines show the 95% limits of agreement (two standard deviations).

Bias and 95% limit of agreement between SV-TTE and SV-Flotrac at baseline, during PLR, before VE and after VE are shown in Figure 5. Percentage error between CO-TTE and CO-Flotrac at baseline, during PLR, before VE and after VE were 25%, 27%, 30% and 29%, respectively.

## Discussion

This study demonstrates that PLR-induced changes in SV-Flotrac are able to predict fluid responsiveness in spontaneously breathing patients without vasoactive support. However, changes in SV-Flotrac were observed after a longer delay than changes in SV-TTE.

To our knowledge, this is the first clinical study evaluating this issue. The accuracy of Vigileo™/Flotrac™ to assess CO has been tested in numerous settings with various results [23-27]. During cardiac surgery and using the second-generation device, Mayer and colleagues showed a good agreement with intermittent pulmonary artery thermodilution [27]. In contrast, it seems that the Vigileo™ does not accurately determine absolute CO values in the event of profound systemic vasodilation (septic shock or liver transplantation) and in unstable patients [23,24,26].

Recently published studies investigating the ability of the Vigileo™ system to track changes in SV showed discordant results. Sakka and colleagues studied 24 mechanically ventilated patients with sepsis and found that the Vigileo™ was unable to track changes in SV induced by an increase in norepinephrine dosage. Patients exhibited very low SVR at baseline, which increased significantly after the intervention [26]. Biancofiore and colleagues studied 29 patients undergoing liver transplantation with very low SVR and showed that changes in CO-Flotrac did not correlate well with changes in CO measured by pulmonary artery catheter [28]. In contrast, it has been demonstrated that in patients with sub-normal SVR, the Vigileo™ is able to track changes in SV induced by mechanical ventilation, VE or body positioning [9,29,30]. In the present study, SVR at baseline were sub-normal and PLR induced significant changes in SVR only in Rs.

Three patients did not exhibit changes in SV-Flotrac following PLR whereas they were Rs to VE. In these patients, SV-TTE increased by more than 13% during PLR. The absence of reactivity of the device probably stems from the algorithm and not from the PLR maneuver. Two of the three patients presented severe sepsis and their SVR was low (573 and 790 dyn/sec/cm^5^). This is in accordance with previously published data and underlines that changes in SV-Flotrac induced by PLR may be somewhat unreliable in patients with very low SVR. A third version of the device has recently been designed to improve SV estimation in septic patients.

In all patients included in the study, the effect of PLR on SV-TTE occurred in the first 90 seconds whereas it occurred in the first 120 seconds for the SV-Flotrac. This may be due to the algorithm of the device which performs SV calculation on the most recent 20 seconds and the k calibration every one minute. This has to be taken into account in clinical practice.

PLR induces a gravitational transfer of blood from the lower part of the body to the intrathoracic compartment and increases cardiac preload [16]. Several types of PLR have been proposed to test fluid responsiveness [12-15,31]. The final position induced by PLR was similar (lower limbs elevated at 45° and trunk in supine position), but the baseline positions were different. The trunk may be elevated at 45° (semi-recumbent), at 30° or supine. It has been recently shown that PLR using the semi-recumbent position at baseline induced a greater increase in cardiac preload and in cardiac index than PLR using the supine position as baseline and that it is preferable for assessing fluid responsiveness [10].

Our study has some limitations. First, the sample was small and may limit the interpretation of the results. Second, only seven septic patients were included and none of the patients received vasopressive drugs. The findings cannot be extrapolated to patients with severe sepsis and receiving vasopressive support. Third, we used SV assessed by transthoracic echocardiography as reference. Transthoracic echocardiography has its inherent limitations but we took care to obtain interpretable measurements: the VTIAo was averaged over five consecutive measurements and four patients were excluded for unsatisfactory cardiac echogenicity. Finally, patients were defined as Rs to VE if SV-TTE increased by 15% or more. This threshold was chosen by reference to previous studies [6,8,12].

## Conclusions

Our findings suggest that in spontaneously breathing patients with subnormal SVR and without vasoactive support, changes in SV-Flotrac induced by PLR correlate with changes in SV-TTE and are able to predict fluid responsiveness, and that the maximal change in SV-Flotrac during PLR occurred in the first 120 seconds. Other studies are necessary to test the accuracy of the Vigileo™ to track changes in SV induced by a PLR maneuver in patients with low SVR.

## Key messages

• PLR-induced changes in SV obtained with SV-TTE and SV-Flotrac are correlated.

• PLR-induced changes in SV-Flotrac are able to predict fluid responsiveness in spontaneously breathing patients without vasopressive support.

• The effect of PLR occurred in the first 120 seconds for the SV-Flotrac and in the first 90 seconds for the SV-TTE.

## Abbreviations

CI: confidence interval; CO: cardiac output; CO-Flotrac: cardiac output obtained with Vigileo device; CO-TTE: cardiac output obtained with transthoracic echocardiography; CVP: central venous pressure; HR: heart rate; MAP: mean arterial pressure; NRs: non-responders; PLR: passive leg raising; ROC: receiver operating curve; Rs: responders; SD: standard deviation; SV: stroke volume; SV-Flotrac: stroke volume obtained with Vigileo device; SV-TTE: stroke volume obtained with transthoracic echocardiography; SVR: systemic vascular resistance; VE: volume expansion; VTIAo: velocity time integral of aortic blood flow.

## Competing interests

The authors declare that they have no competing interests.

## Authors' contributions

MB conceived and designed the study. MB performed all transthoracic echocardiography. MB, LV, PS, LP and VC performed data acquisition. MB, PR and FS participated in the data analysis and interpretation of the results. MB and FS were involved in the statistical analysis and wrote the paper. All authors read and approved the final manuscript.
